# Law Providing for Sterilization of Epileptics in Charitable Institutions Not Constitutional

**Published:** 1914-06

**Authors:** 


### Law Providing for Sterilization of Epileptics in Charitable Institutions Not Constitutional.

The Supreme Court of New Jersey holds unconstitutional, as to epileptics in charitable institutions, the statute of 1911 of that State, authorizing and providing for the sterilization of feebleminded (including idiots, imbeciles, and morons), epileptics, rapists, certain criminals, and other defectives. The court says that the application of the statute to criminals, did not concern it in this case, as the prosecutrix was an epileptic, an unfortunate person, but not a criminal.

The board of examiners created by the statute made an order which, after reciting that Alice Smith was an epileptic inmate of a State charitable institution, that procreation by her was inadvisable, and that there was no probability that her condition would improve to such an extent as to render procreation by her advisable, ordered that an operation of salpingectomy be performed on her. She fell within the classification of the statute in that she was an inmate of the State Village of Epileptics, of which she had been an inmate since 1902. She had had no attack of the disease for the five years last past.

The court after stating that the statute is broad enough to authorize an operation for the sterilization of an epileptic, discusses the nature and dangers of such an operation and says it must be performed by force, at least to the extent of the production of anesthesia which would destroy all liberty of will or action, and that it would threaten the life, and certainly the liberty, of the individual in a manner forbidden by both the State and Federal Constitutions, unless it were a valid exercise of the police power.

The question presented is, therefore, not one of those constitutional questions that are primarily addressed to the Legislature, but a purely legal question of police power, which is a matter for determination by the courts. The police power is defined as the power in the Legislature to enact and enforce whatever regulations are in its judgment demanded for the welfare of society at large in order to secure or guard its order, safety, health, or morals. The case in hand raises the very important novel question whether it is one of the attributes of government to essay the theoretical improvement of society by destroying the function of procreation in certain of its members who are not malefactors against its loss. The courts say that the feeble-minded and epileptics are not the only persons in the community whose elimination as undersirable citizens would, or might in the judgment of the Legislature, be a distinct benefit to society, and if the enforced sterility of this class be a legitimate exercise of governmental power, a wide field of legislative activity and duty is thrown open to which it would be difficult to assign a legal limit. Passing the discussion of these questions, the court bases its decision in this case on the ground that the statute makes an unconstitutional classification with reference to epileptics, in that it applies only to epileptics who are "inmates confined in the several charitable institutions in the counties and State." The court states that a further objection is that the law, when taking into consideration the magnitude of the purpose in view, which is nothing less than the artificial improvement of society at large, is singularly inapt, inasmuch as it does not apply to the much larger class of epileptics who are not confined in the county and State institutions and are vastly more exposed to the temptation and opportunity of procreation, to the much greater injury to society. The suggestion that the classification might be sufficient if the scheme of the statute were to turn the sterilized inmates of the institutions loose in the community, the court dismisses as not worthy of serious consideration.—*Journal A. M. A.*

Why quibble over technicalities and man-made laws when the rights of the unborn are involved? When will educated society realize that the laws of biology (life) are the basis of all laws and customs that affect the health and happiness of the individual. When man-made statutes conflict with well established biological truths they must give way, especially when such laws are in the nature of technicalities, verbiage, etc. There must be a readjustment of the morals of society before these stumbling blocks give way to progress. Education is the great leveler of human error.

